# The Ticket System

**Published:** 1892-02-20

**Authors:** 


### THE TICKET SYSTEM.

The Committee of the Hospital for Sick Children, Great Ormond Street, have long been famous for the deep personal interest they take in the welfare of their institution. After long and careful consideration, the Committee have determined to consult the Governors and the Subscribers with a view to the abolition or reform of the present system of letters of admission. It is pointed out that ninety per cent. of the Governors' and Subscribers' letters are at present unused, and that the interests of the little patients demand the promptest admission to and treatment in the hospital. It is proposed to abolish in-patient letters altogether, and to pass every case, as far as possible, through the out-patient department, the Governors receiving recommendations which entitle the patients to such relief, whether in or out, as the circumstances of each case demand, precedence to be given as between cases of equal urgency to children who hold Subscribers' recommendations. We agree with the Committee that such a modification of their system would not only be more advantageous to the hospital, but more satisfactory to Governors and Subscribers than the present one. It has been determined to submit the new proposals to a special court which, we have little doubt, will cordially adopt the modifications suggested. There can be no doubt that the Committee's proposals will prove a boon to the patients, and redound in every way to the credit of the Hospital for Sick Children, which already stands so deservedly high in public estimation.

